# Identification of biomarkers and target drugs for melanoma: a topological and deep learning approach

**DOI:** 10.3389/fgene.2025.1471037

**Published:** 2025-03-03

**Authors:** Xiwei Cui, Jipeng Song, Qingfeng Li, Jieyi Ren

**Affiliations:** ^1^ Research Center of Plastic Surgery Hospital, Chinese Academy of Medical Science and Peking Union Medical College, Beijing, China; ^2^ Key Laboratory of External Tissue and Organ Regeneration, Chinese Academy of Medical Sciences and Peking Union Medical College, Beijing, China; ^3^ Comprehensive Ward of Plastic Surgery, Plastic Surgery Hospital, Chinese Academy of Medical Sciences and Peking Union Medical College, Beijing, China; ^4^ Department of Plastic and Reconstructive Surgery, Shanghai Ninth People’s Hospital, Shanghai Jiao Tong University School of Medicine, Shanghai, China

**Keywords:** melanoma, tumor, immune, next-generation sequencing (NGS), topology, drug screening, plastic surgery

## Abstract

**Introduction:**

Melanoma, a highly aggressive malignancy characterized by rapid metastasis and elevated mortality rates, predominantly originates in cutaneous tissues. While surgical interventions, immunotherapy, and targeted therapies have advanced, the prognosis for advanced-stage melanoma remains dismal. Globally, melanoma incidence continues to rise, with the United States alone reporting over 100,000 new cases and 7,000 deaths annually. Despite the exponential growth of tumor data facilitated by next-generation sequencing (NGS), current analytical approaches predominantly emphasize single-gene analyses, neglecting critical insights into complex gene interaction networks. This study aims to address this gap by systematically exploring immune gene regulatory dynamics in melanoma progression.

**Methods:**

We developed a bidirectional, weighted, signed, and directed topological immune gene regulatory network to compare transcriptional landscapes between benign melanocytic nevi and cutaneous melanoma. Advanced network analysis tools were employed to identify structural disparities and functional module shifts. Key driver genes were validated through topological centrality metrics. Additionally, deep learning models were implemented to predict drug-target interactions, leveraging molecular features derived from network analyses.

**Results:**

Significant topological divergences emerged between nevi and melanoma networks, with dominant functional modules transitioning from cell cycle regulation in benign lesions to DNA repair and cell migration pathways in malignant tumors. A group of genes, including AURKA, CCNE1, APEX2, and EXOC8, were identified as potential orchestrators of immune microenvironment remodeling during malignant transformation. The deep learning framework successfully predicted 23 clinically actionable drug candidates targeting these molecular drivers.

**Discussion:**

The observed module shift from cell cycle to invasion-related pathways provides mechanistic insights into melanoma progression, suggesting early therapeutic targeting of DNA repair machinery might mitigate metastatic potential. The identified hub genes, particularly AURKA and DDX19B, represent novel candidates for immunomodulatory interventions. Our computational drug prediction strategy bridges molecular network analysis with clinical translation, offering a paradigm for precision oncology in melanoma. Future studies should validate these targets in preclinical models and explore network-based biomarkers for early detection.

## 1 Introduction

Melanoma is a highly malignant cancer originating from melanocytes, notorious for its rapid spread and high mortality rates. Even tumors just a few millimeters in size can be fatal, making it one of the most aggressive forms of cancer. Melanomas primarily occur on the skin (over 90% of all melanoma diagnoses), with mucosal and uveal melanomas being less common (<1–5% of diagnoses, varying by region). There are also rare cases in children with neurocutaneous melanocytosis where melanomas occur in the central nervous system (CNS) (Bittencourt et al., 2000; Elder et al., 2020; Neale et al., 2022). Over the past half-century, the incidence of cutaneous melanoma has been steadily rising globally, surpassing the incidence rates of other cancers (Welch et al., 2021; Sung et al., 2021). The incidence is highest among non-Hispanic white patients, primarily attributed to ultraviolet (UV) exposure. Individuals with skin of color have lower incidence rates but significantly lower survival rates, mainly due to delayed diagnosis, insufficient patient education, and limited treatment options (Brunsgaard et al., 2023). According to a 2021 U.S. statistic, an estimated 106,110 new cases of melanoma were diagnosed, and 7,180 deaths occurred due to the disease (Siegel et al., 2021). Additionally, patients’ survival and quality of life are significantly impacted, with over one-third of melanoma patients reporting clinically significant levels of distress (Cornish et al., 2009; Rosenberg et al., 2021). Survival rates vary significantly depending on the tumor site, as the primary treatment for melanoma is surgical excision. According to the SEER (Surveillance, Epidemiology, and End Results) database, the 5-year survival rate for localized melanomas amenable to early surgical intervention is nearly 100%. In contrast, the 5-year survival rates for regional (involving regional lymph nodes) and distant (metastatic) melanomas are 74.8% and 35%, respectively (Patel V. R. et al., 2023; National Institutes of Health, 2024). Understanding the molecular networks of melanoma is crucial for improving patients’ survival and quality of life.

For a long time, treatment options for melanoma were highly limited, resulting in extremely low survival rates for advanced-stage patients. However, immune checkpoint inhibitors (ICI) and target medicines have made significant progress in improving survival rates, particularly for those with inoperable advanced melanoma (Patel S. P. et al., 2023). Before 2010, only dacarbazine chemotherapy and high-dose interleukin-2 (IL-2) were approved by the U.S. Food and Drug Administration (FDA) for the treatment of metastatic melanoma (Lo and Fisher, 2014). These treatments had limited efficacy and significant side effects. Currently, therapies targeting CTLA-4, PD-1, and PD-L1 have increased the 5-year survival rate for patients with advanced melanoma from less than 5% to 30%–40% (Hamid et al., 2019; Spagnolo et al., 2019). This success has established ICIs and target therapies as first-line treatments for melanoma and other cancer types. However, 40%–60% of melanoma patients do not achieve significant therapeutic effects, and many responders experience tumor recurrence (Kalaora et al., 2022). Low response rates and frequent treatment resistance impede further improvements in therapeutic outcomes. Therefore, a more comprehensive understanding of the immunomolecular mechanisms of melanoma is necessary. The immune environment of melanoma is highly complex, and further interpretation of related experimental data is urgently required.

In the past decade, the cost reduction and widespread adoption of new technologies have led to an explosive growth in sequencing data. This has significantly enhanced our understanding of the molecular regulatory networks involved in various diseases. However, our methods for interpreting sequencing data have not kept pace. Current stratification methods, which are based on gene expression differences, often focus on single genes. While this reductionist approach can simplify the identification of key genes associated with disease risk, it fails to capture the complexity of disease processes, which are regulated by intricate networks of multiple genes. Ethical constraints in research further complicate our efforts, as it is often challenging to obtain sequencing data from different pathological stages within the same individual, such as multiple samples during the progression from nevi to melanoma. Isolated time-point data hinder our ability to observe dynamic changes in molecular regulatory networks and their interrelationships. In contrast, directed topological network models are well-suited for describing these complexities and have shown promising results in understanding disease mechanisms.

In this study, we constructed a bidirectional, weighted, signed, and directed topological immune gene regulatory network of human cutaneous melanoma. We applied and adapted an ecological model, which was originally used in ecological and biological research. This model provided new insights into tree growth characteristics and gut microbiome statistics. Within this topological network, we conducted a comprehensive analysis of transcriptome sequencing data to describe the immune gene regulatory networks that may influence melanoma formation. We compared the molecular regulatory network differences between benign nevus and melanomas. At the same time, we also introduced directional topological homology theory to compare differences between various topological networks. Using this approach, we characterized previously unknown gene interactions that could better represent biomarkers of melanoma. Additionally, we used deep learning models to make drug-target interaction (DTI) predictions, offering new insights for clinical drug selection for melanoma treatment.

## 2 Methods

### 2.1 Real-world datasets

A cross-sectional cohort study was conducted to collect transcriptome sequencing data from a series of benign melanocytic nevi and primary melanoma samples (Kunz et al., 2018). This dataset comprises 23 benign melanocytic nevi samples and 57 primary melanoma samples. The thickness of the harvested primary melanoma tumors, measured according to the Breslow principle—a widely used metric for evaluating melanoma invasion—ranged from 0.40 to 3.86 mm. All analyses were approved by the local ethics committee and conducted in accordance with the principles outlined in the Declaration of Helsinki. Detailed descriptions of the cohort study design, sampling strategy, and transcriptome sequencing have been previously published (Kunz et al., 2018).

Sequencing data were annotated using Illumina platform information and Ensembl Gene IDs, resulting in expression data for 33,897 genes. From these, 3,272 immune-related genes were identified based on annotations from InnateDB, currently the most comprehensive database for annotating immune genes and protein functions (Breuer et al., 2013). Using the expression data of these genes, we reconstructed topological networks to focus on analyzing the fluctuations in immune networks during melanoma progression. Differentially expressed genes (DEGs) were analyzed using the limma package. Figures were created using R packages, including ggplot2 and tidyHeatmap. Enrichment analysis was conducted using the clusterProfiler package and Metascape (Zhou et al., 2019).

### 2.2 Topological network design

**FIGURE 1:**
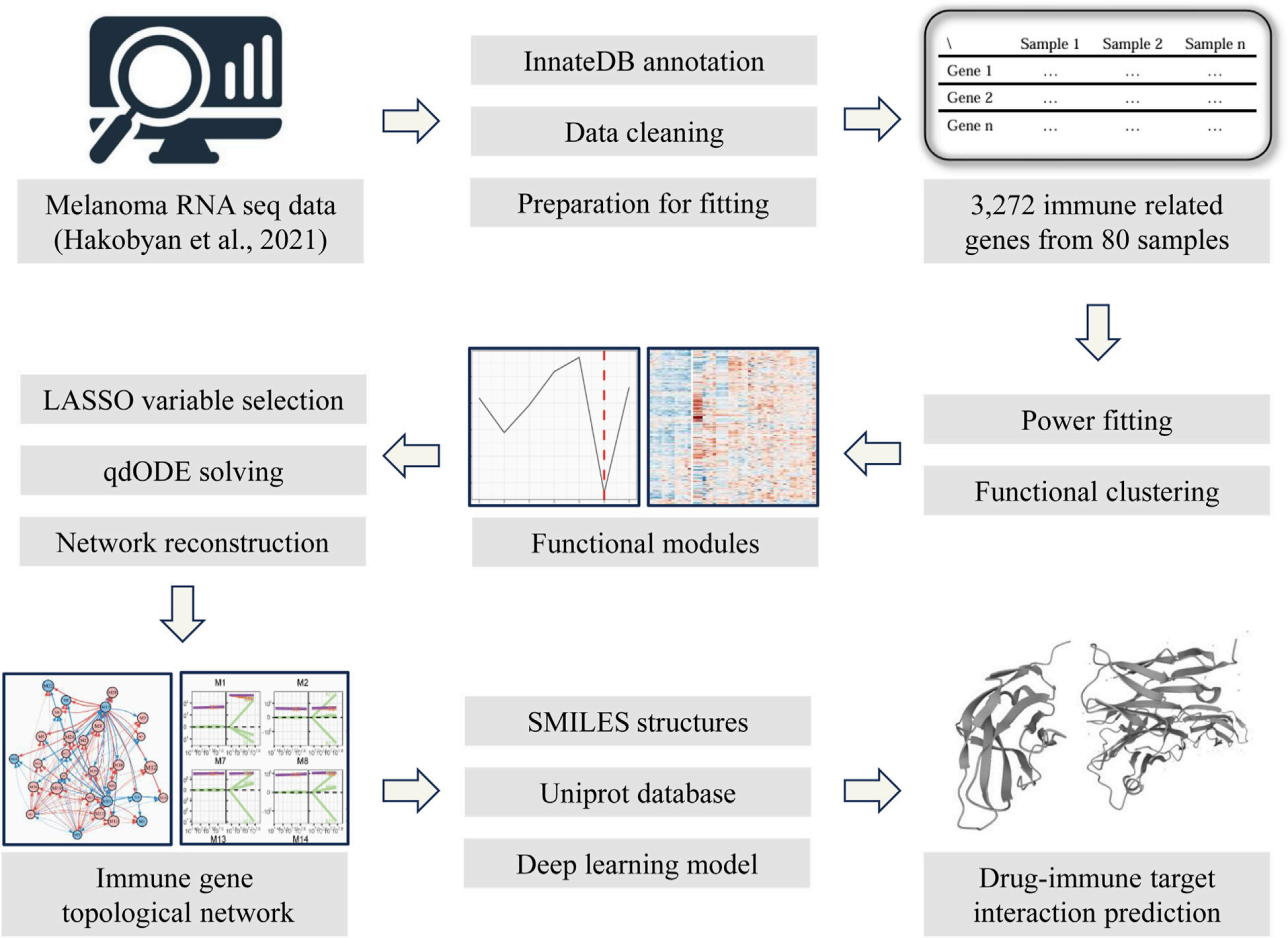
The methodological workflow of this study.

The reconstruction of the immune topological network was based on the idopNetwork model proposed by Professor Rongling Wu’s team (Chen et al., 2019; Dong et al., 2023) and subsequently adapted for our study. This model is a mathematical framework rooted in ecological theory, initially designed to describe the regulatory interactions among various species and populations within an ecosystem. While population change curves can be observed in an ecosystem, the direct or indirect regulatory relationships among populations remain obscured (e.g., cooperation/symbiosis, inhibition/predation, or independence). Similarly, in the study of gene expression patterns within an organism, comparable challenges arise. In this research, we treated each sample as an ecosystem and each gene (or cluster) as a population within that ecosystem, using a directed topological network to mathematically represent their expression patterns (Figure 1). This approach allowed us to infer the bidirectional dynamic regulatory relationships between each gene (or cluster) and their associations with the overall gene expression background from static sequencing data.

### 2.3 Gene expression patterns fitting and functional clustering

Assume we have m RNA-seq samples, stratified based on clinical data and patient information such as age, sex, Breslow thickness, BRAF mutation, NRAS mutation, and pathological verification of inflammatory infiltration. Additionally, samples are classified into benign (melanocytic nevi) and malignant (melanoma) groups based on pathological diagnosis. For each sample, the expression levels of n genes are measured, and the gene IDs are consistent across all samples. Consider $g_{ij}$ as the abundance of gene i in sample $\left(with\;i=1,\ldots ,m\;and\;j=1,\ldots ,n\right)$ . Each sample can be viewed as an ecosystem made up of m interacting genes. The total expression of all genes in a sample is termed the habitat index (HI), denoted as:

$$Hi=\sum_{j=1}^{m}g_{ij}$$

This index reflects the sample’s ability to support the simultaneous expression of all its genes. Under this framework, $g_{ij}$ and $H_{i}$ establish a part-whole relationship across samples, meaning that $g_{ij}$ can be modeled as a function of Hi, represented as $g_{j}\left(Hi\right)$ . This conceptual framework forms the basis for reconstructing regulatory networks in benign nevi and melanoma samples. The expression level of a single gene and the overall expression level generally follow the part-whole relationship described by the allometric scaling law (Zhou et al., 2021; Baller et al., 2019). Therefore, we attempt to quantify the mathematical relationship between the overall gene expression background and a specific gene using a power function equation, represented as:

$$g_{ij}=\alpha_{j}H_{i}^{\beta_{j}}$$

where $\alpha_{j}$ is the intercept constant for gene j , and $\beta_{j}$ is its scaling exponent. Together, they determine the shape of the fitted power function curve, reflecting the variation of gene j as the overall expression abundance i changes.

Due to the large number of genes, directly calculating the interactions among all genes would complicate data interpretation. Studies suggest that in constructing network models, the number of nodes should be limited to a manageable level to satisfy Dunbar’s law, ensuring modeling accuracy (Lehmann and Dunbar, 2009). Based on the similarity of the allometric scaling curves fitted to different genes, we used a mixture functional clustering algorithm to cluster the genes (Kim et al., 2008). This method combines *k*-means clustering with the expectation-maximization algorithm and the simplex algorithm to optimize model parameters. In this algorithm, the posterior probability of each gene belonging to each module is calculated to determine its most likely assignment. Additionally, we use information criteria (Akaike Information Criterion, AIC, and Bayesian Information Criterion, BIC) to assess whether the clustering result is optimal within the given range. This clustering strategy allows us to reconstruct multi-layered topological networks to simplify the model. Compared to analyzing the interactions of all genes directly, the number of genes in each module is much smaller, and the genes within each module are likely more closely related. Moreover, by calculating the overall expression abundance of each module, we can directly compute the regulatory relationships between modules to identify the most critical immune gene modules.

### 2.4 Inferring gene interactions based on evolutionary game theory

The inter-regulatory relationships between genes can be explained through the concepts of evolutionary game theory. In a system, the abundance of different genes is determined by their intrinsic expression levels as well as their mutual regulatory interactions with other genes. This complex regulatory network will eventually reach a Nash equilibrium, typically described as follows:

$$s_{i}^{*}\in {\arg\;\max}_{s_{i}\in S_{i}}u_{i}\left(s_{i},s_{-i}^{*}\right)$$

In this formula, $s_{i}^{*}$ represents the optimal strategy for player i , which maximizes their utility $u_{i}$ given the strategies $s_{-i}^{*}$ of all other players. This state occurs when no player can improve their payoff by unilaterally changing their strategy, signifying a stable and balanced outcome in the network. In the context of gene expression, this means that the expression levels of different genes stabilize under a given gene expression environment. By combining evolutionary game theory with a predator-prey model through allometric scaling laws, we can construct a quasi-dynamic system of ordinary differential equations (qdODE).

$$g_{j}^{\prime }\left(H_{i}\right)=Q_{j}\left(g_{j}\left(H_{i}\right);\emptyset_{j}\right)+\sum_{j^{\prime }\neq j}Q_{j}\leftarrow j^{\prime }\left(g_{j^{\prime }}\left(H_{i}\right);\emptyset_{j}\leftarrow j^{\prime }\right)$$

On the left side of the differential equation, the time derivative is replaced by the HI derivative, representing the rate of change of gene j ’s expression level under the influence of the environment $H_{i}$ . On the right side, the expression level of a gene is determined by both independent and dependent components. $Q_{j}\left(g_{j}\left(H_{i}\right);\emptyset_{j}\right)$ captures the intrinsic regulatory effects on gene j ’s expression. $\sum_{j^{\prime }\neq j}Q_{j}\leftarrow j^{\prime }\left(g_{j^{\prime }}\left(H_{i}\right);\emptyset_{j}\leftarrow j^{\prime }\right)$ represents the regulatory effects of other genes $j^{\prime }$ on gene j ’s expression. This approach allows us to solve the differential equation using allometric scaling curves and Legendre polynomials, ultimately revealing the intrinsic expression capabilities of each gene as well as the interaction relationships among different genes. Based on the results from this equation, we can further construct a directed topological network.

### 2.5 Variable selection and network reconstruction

Based on empirical evidence and extensive experimental data, it has been confirmed that in a gene regulatory network, it is impossible for a gene to have significant regulatory interactions with all other genes. Connecting all nodes would make the network structure highly vulnerable to random perturbations, causing noise to be mistaken for interactions between nodes. This implies that computing the interactions between all genes is unnecessary and unscientific when deriving a gene network model. To identify the most significant dependent components, a regression model based on ridge regression and lasso regression has been introduced, typically described as follows:

$$\min\left\{{\left\|y-Xw\right\|}_{2}^{2}\right.+\left.\lambda {\left\|w\right\|}_{2}^{2}\right\}$$

$$\min\left\{{\left\|y-Xw\right\|}_{2}^{2}\right.+\left.{\lambda \left\|w\right\|}_{1}\right\}$$

where y is the target variable vector, X is the feature matrix to be selected, w is the regression coefficient vector, and λ is the regularization parameter. For each target variable, we specifically minimize these two objective functions in sequence to select the appropriate interaction pairs. Simultaneously, a non-parametric approach is introduced to optimize model construction. The non-parametric method determines regression coefficients flexibly without relying on a specific functional form, which helps capture complex relationships within the data. Additionally, the penalty parameter is determined by BIC or extended BIC to balance model complexity and fit, while a weighting function is used to accelerate algorithm convergence, thereby improving computational efficiency. By solving the regression problem, the variables/genes that most significantly affect gene j are selected for re-solving the qdODE, facilitating network reconstruction. Following these steps, we reconstructed a directed topological network for melanoma immune genes using the selected genes/modules and their qdODE equations. Each gene/module’s independent component is treated as a node, while the corresponding dependent components are treated as edges. These edges are labeled as positive or negative to indicate promoting or inhibiting relationships and are weighted by the absolute value of the dependent component to represent the regulation strength.

As a result, the model is a two-layer directed topological network. The first layer represents the regulatory interactions between different modules, while the second layer represents the regulatory network within each module. By comparing the differences between these regulatory networks, we may explore the differences in immune regulation networks between nevi and melanomas.

### 2.6 Drug prediction based on deep learning

Based on the results of the topological network reconstruction, we identified genes as potential therapeutic targets. The amino acid sequences corresponding to these genes were obtained from the UniProt database (https://www.uniprot.org/). We then searched various drug and compound databases to obtain the SMILES chemical structures of drugs that are “marketed,” in “Phase I/II/III clinical trials,” “pre-registered,” or “registered.” Using the DeepPurpose model, we performed drug–target interaction (DTI) predictions (Huang et al., 2021). This model, based on deep learning, combines previously proposed amino acid and compound encoders in various ways to predict binding affinity, encompassing a total of 15 prediction modes. The final data displayed the top five results from each prediction mode.

## 3 Results

### 3.1 Abundance analysis of individual and overall immune genes

In our study, a total of 3,272 immune-related genes from 80 samples were analyzed. The clinical information of the patients from whom the samples were derived varied, including factors such as age, sex, tumor (benign or malignant) infiltration depth, and BRAF and NRAS mutation status. Samples were stratified into two groups based on pathological diagnosis: melanocytic nevi (n = 23) and melanoma (n = 57). Previous studies often identified several differentially expressed genes as marker or potential target genes. However, changes in expression levels do not directly reflect the function of each gene, prompting the development of new mathematical modeling methods.

**FIGURE 2:**
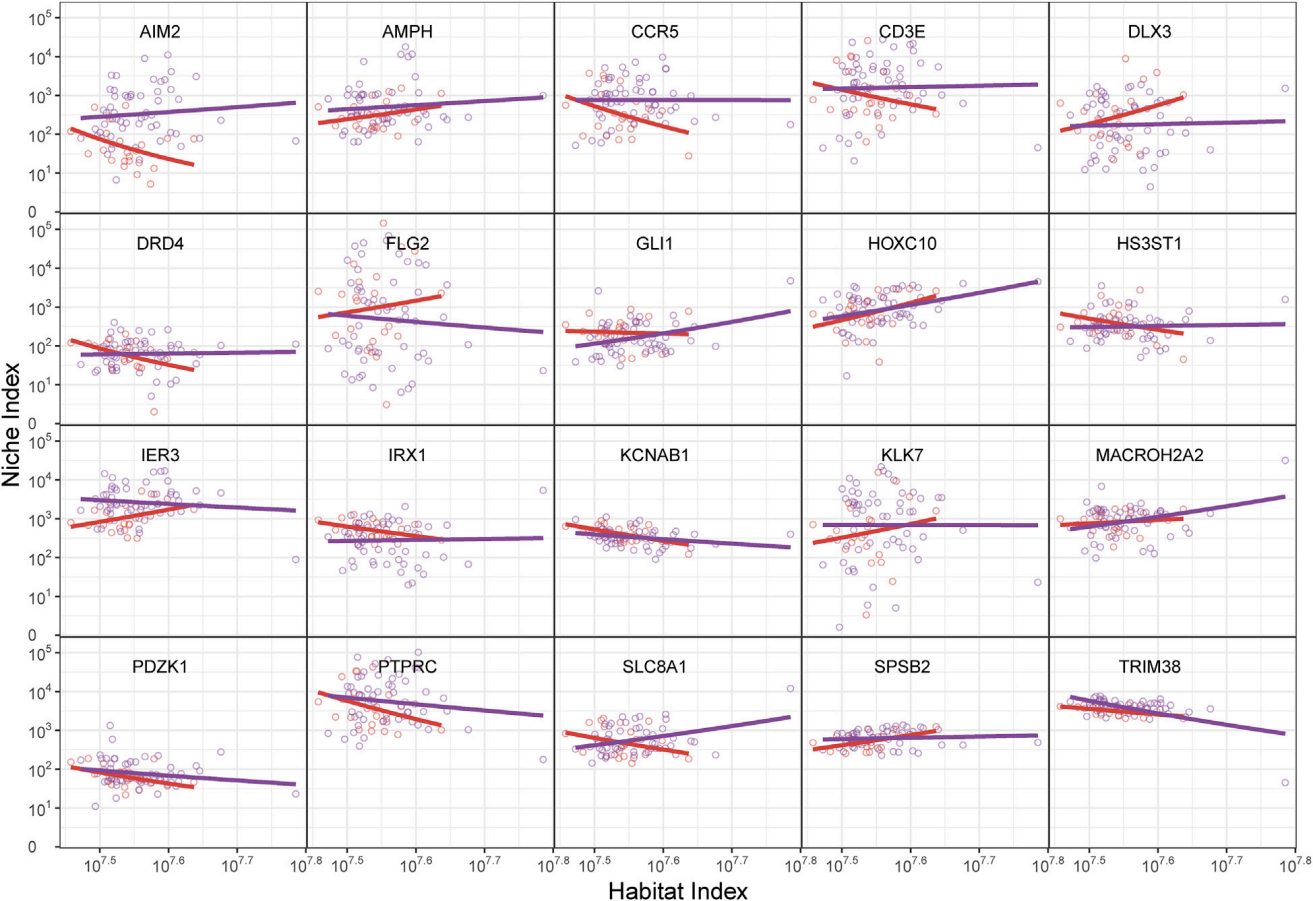
The relationship between gene abundance (niche index, NI) and total gene abundance (habitat index, HI) in randomly selected genes from nevi and melanoma, shown by the fitting of the allometric scaling equation. The HI is on the *x*-axis and the NI is on the *y*-axis. The dots represent the NI and HI levels of specific genes within individual samples, while the curves depict functional fittings derived through allometric scaling laws. The red curve represents the benign group, and the purple curve represents the malignant group. The shape of the curves is determined by their intercept constant and scaling exponent.

We utilized an ecological theory-based approach (idopNetwork) to reconstruct a directed topological network to describe the differences and dynamic patterns of immune activity in the gene networks between melanocytic nevi and melanoma. Mathematically, we defined the Habitat Index (HI) to reflect the overall expression levels of all detected immune genes in each sample. The overall gene expression and the expression level of individual genes followed the allometric scaling law. We attempted to transform gene expression abundance and define it as the Niche Index (NI), fitting it to the allometric scaling equation with the Habitat Index. The scaling-law equation demonstrated robust capacity to capture the nonlinear dynamics of gene expression level variations in response to habitat instability (HI) changes, as evidenced by the strong agreement between model predictions and empirical data (Figure 2, dots vs fitted curves). The genes were randomly selected by the algorithm to verify the universality of the successful fit. Genes displayed divergent regulatory behaviors: HOXC10 showed progressive upregulation with increasing HI, while PTPRC and TRIM38 exhibited significant downregulation. The directionality of these nonlinear interaction (NI) trajectories reflects fundamental biological adaptations - upward curves indicate transcriptional amplification under environmental fluctuation, whereas downward patterns suggest targeted pathway suppression during gene background (habitat) destabilization.

Notably, our analysis revealed pronounced intergroup disparities in NI expression patterns, particularly exemplified by genes including AIM2, FLG2, and SLC8A1. Furthermore, systematic evaluation of allometric scaling parameters ( α , scaling exponents; β , proportionality constants) identified 2,166 genes demonstrating statistically significant alterations, defined as α deviations exceeding 10-fold or β variations surpassing baseline thresholds. We calculated the residuals for each gene and plotted them against the predicted values. The independence of these residuals indicated the statistical robustness of the power fit. Furthermore, we noticed that the HI range for nevus samples was lower than that for the melanoma group, which might indicate differences in gene expression backgrounds between the two. However, a higher HI range should not be simply interpreted as pointing to melanoma risk, and further topological network reconstruction is necessary.

### 3.2 Functional clustering of the melanoma immune network

**FIGURE 3:**
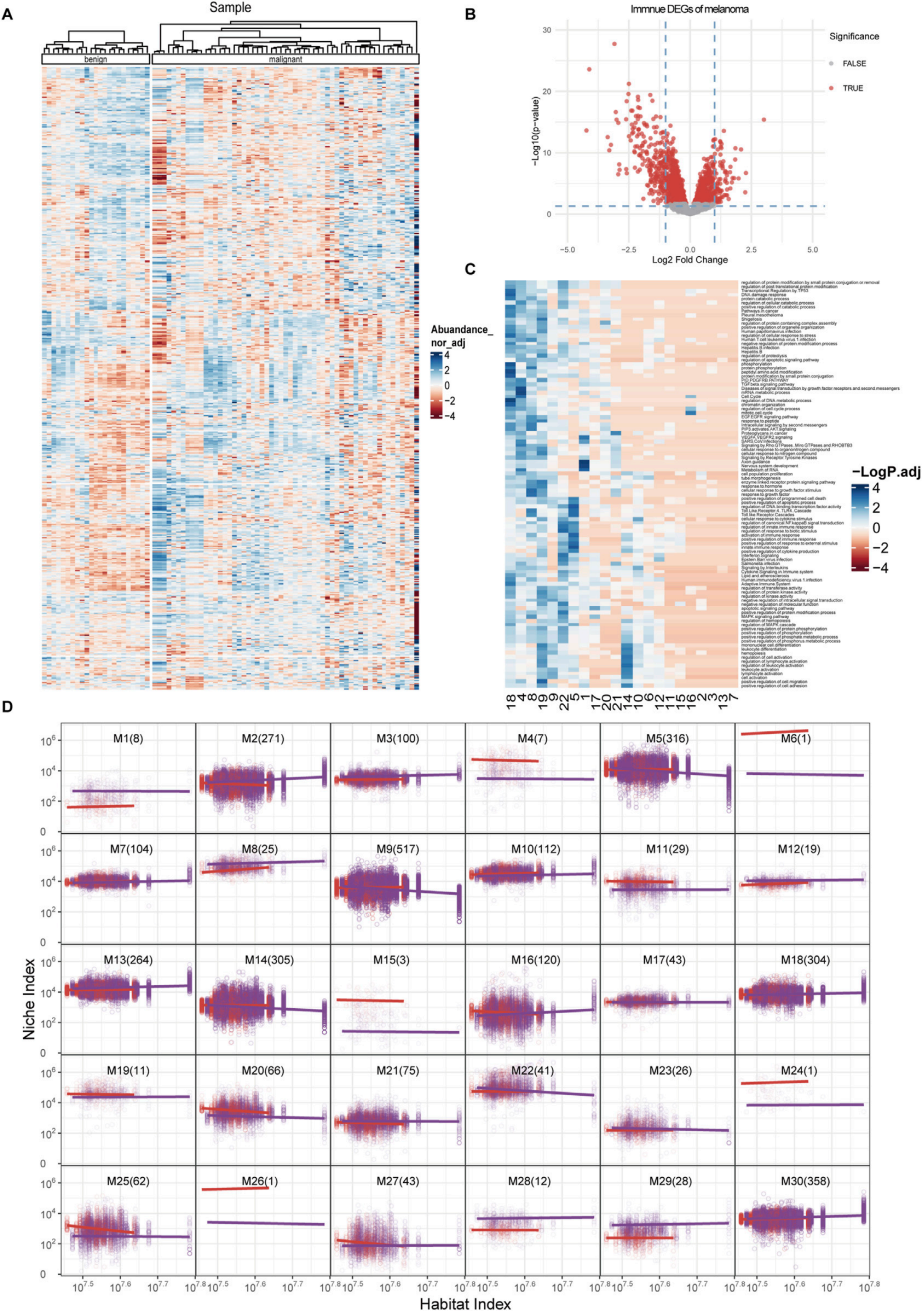
Functional clustering and module allometric scaling fit of immune networks. **(A)** Heatmap of the 3,272 immune-related genes included in the analysis, grouped by their clusters and by benign or malignant status. **(B)** Volcano plot of DEGs among all immune-related genes. **(C)** Top enriched functions in each module. *X*-axis, different modules derived from functional clustering process. *Y*-axis, enriched functions. **(D)** Allometric scaling curves for each clustered modules, with the habitat index on the *x*-axis and the niche index on the *y*-axis. The numbers in parentheses indicate the quantity of genes contained within each module. The dots represent the NI and HI levels of specific genes within individual samples, while the curves depict functional fittings derived through allometric scaling laws. The red curve represents the benign group, and the purple curve represents the malignant group.

To further elucidate the topological patterns within gene networks, we implemented functional clustering following the theoretical framework established by Kim et al. (2008). This methodological choice was particularly appropriate since our data transformation had already converted gene expression profiles into allometric scaling equations describing the relationship between individual genes and the systemic background. Unlike conventional unsupervised clustering methods, this functional approach inherently accounts for the scale-dependent biological constraints embedded in the transformed data. The clustering optimization process was systematically guided by information-theoretic criteria, with Bayesian Information Criterion (BIC) and Akaike Information Criterion (AIC) being employed to determine optimal cluster configurations (Supplementary Figure S1A). This dual-criterion approach ensures both model parsimony and goodness-of-fit while mitigating overfitting risks associated with complex biological datasets. All genes were grouped into 30 functional clusters based on the similarity of their NI change patterns (Figure 3A). This topological modeling approach includes not only the genes with significant differential expression but also those with less pronounced differences between the two sample groups to construct a comprehensive topological network (Figure 3B).

Unlike traditional analysis methods, this approach highlights genes like AIM2 and SLC8A1 from Figure 2, which might not be recognized as significantly differentially expressed genes but show notable intergroup differences in topological analysis. The interaction patterns of these genes within different groups will be reassessed and weighted in subsequent steps. Furthermore, our clustering analysis revealed distinct functional enrichments across different clusters (Figure 3C). The enrichment analysis revealed distinct functional associations for each module. For examples, modules 8, 9, 19, 22, and five demonstrated significant correlation with immune response. Modules 15, 2, 3, 13, and seven were associated with the production and response to inflammatory factors. The activation and migration of immune cells were primarily linked to modules 14 and 19. Module one showed specific relevance to neural cells, while modules 18 and four were associated with cell cycle regulation and phosphorylation activities. We also treated each module as an independent entity to calculate its relationship with the overall gene expression pattern (HI), similar to the analysis we performed to isolated genes. Some modules exhibited significant pattern differences between the two groups, such as M1, M4, M28, and M29, which showed distinct separation curves. In contrast, modules like M5, M20, M25, and M27 demonstrated group-specific expression changes across the HI (Figure 3D).

### 3.3 Melanoma exhibits immune activation and regulation involving multiple hub modules

**FIGURE 4:**
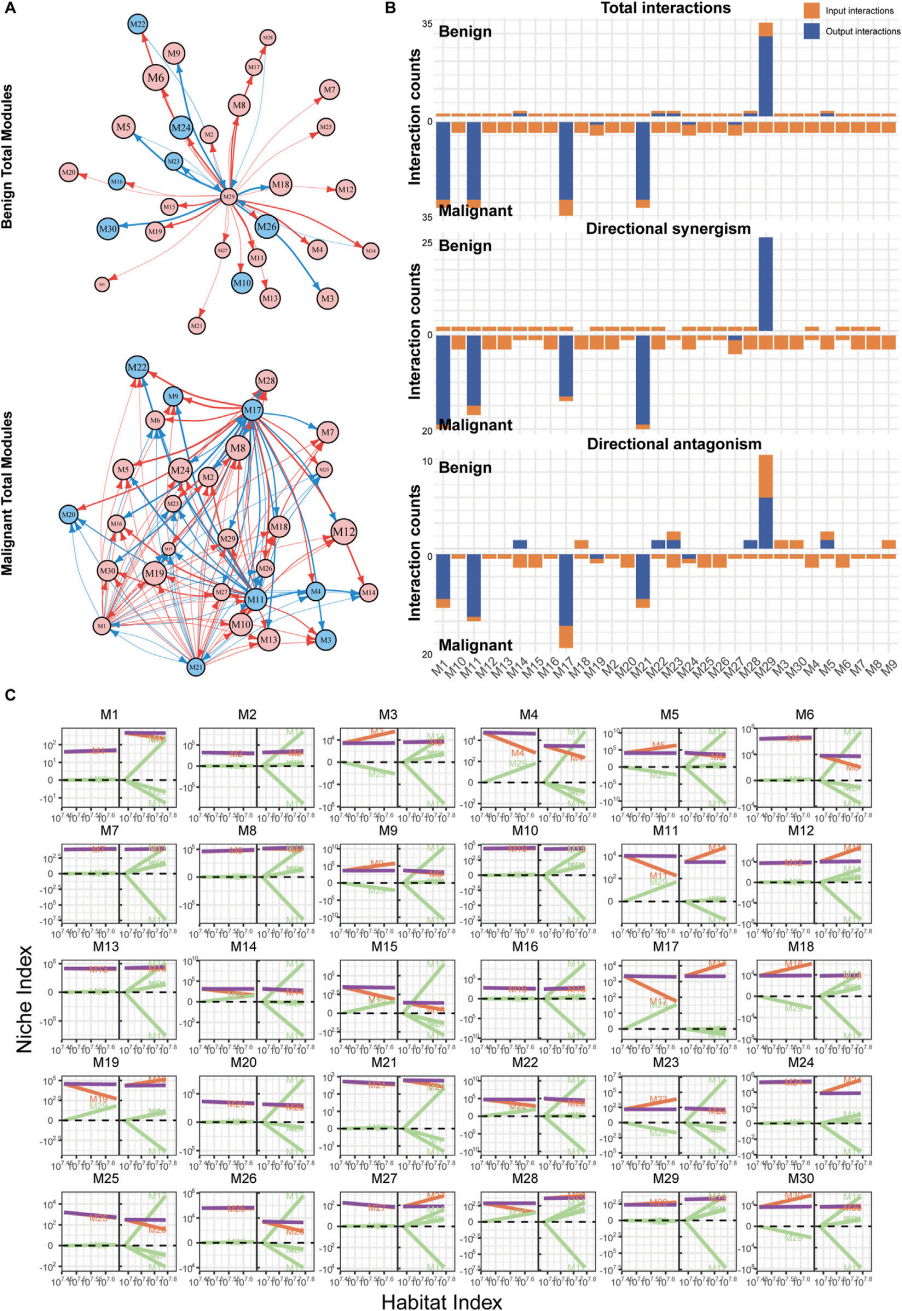
Reconstruction of immune networks in nevi and melanoma. **(A)** Directed topological network of all modules in nevi and melanoma. Each node represents a module, and each edge represents a regulatory relationship. Red arrows indicate promotion, blue arrows indicate inhibition, and the thickness of the curve represents the strength of the regulation. **(B)** Bar chart of the interaction relationships within the two groups’ networks. The *x*-axis represents the modules, and the *y*-axis represents the number of regulations. Orange bars (input) show incoming regulatory interactions from other modules, while blue bars (output) represent outgoing regulations to other modules. **(C)** Decomposition curve of each module’s NI based on the trend of HI changes, obtained from the qdODE equation. The *x*-axis represents the HI, and the *y*-axis represents the NI. The purple curve represents the observed expression, the red curve represents the module’s independent expression ability, and the green curve represents the influence of other modules on this module (dependent component).

Using the qdODE equation, we calculated each module’s independent expression ability and their mutual influences. Based on this data, we reconstructed a directed topological network with modules as nodes (Figure 4A). In the network of nevi, M29 emerged as the most influential and widely affecting module, suggesting that M29 could be the immune hub module in the nevus state. Most modules exhibited either bidirectional or unidirectional synergistic effects with M29, while a few modules, which contained a larger number of genes, inhibited or were inhibited by M29. However, this pattern was dramatically altered in melanoma samples.

Firstly, the connections between modules increased significantly (Figures 3B, 4B). As a result, there was an increase in the number of edges in the network, which heightened the complexity of the topological pattern. The hub module shifted from M29 to M1, M11, M17, and M21 in the melanoma immune network. The regulatory influence of these hub modules on other modules became more intense (Figure 4C), with M17 being the most prominent, followed by M11 (Supplementary Figure S3). Although M1 and M21 had extensive influence, their impact was relatively weaker (Supplementary Figures S2 and S4). The overall regulatory effects on many modules also changed. Some modules that were previously promoted became inhibited (e.g., M11, M17, M19), and some modules that were weakly regulated before exhibited more dependency, such as M6, M12, M24, and M27. These phenomena likely reflect the more complex and intense immune activities in melanoma tissues.

Our comparative analysis of cross-module gene regulation in benign and malignant samples (Figure 4) revealed distinct functional landscapes. qdODE algorithm identified multiple gene-rich modules exhibiting suppressed activity in benign states, with enrichment analysis (Supplementary Figure S1) linking these modules to inflammatory cytokine production, cellular stress response mechanisms, and pro-tumor pathways. This aligns with established mechanisms where benign lesions maintain homeostasis through constrained inflammatory signaling and active suppression of oncogenic processes. In contrast, the melanoma network demonstrated significant functional reconfiguration, with pronounced enrichment of immune-related processes. Specifically, modules governing adaptive immune activation, pro-inflammatory cytokine networks, and immune cell recruitment were robustly activated. This paradoxical amplification of immune-associated functions in malignancy reflects a dynamic interplay wherein tumor cells paradoxically activate immune-modulatory pathways, potentially as a compensatory mechanism to counterbalance malignant progression. The observed dichotomy—suppressed inflammatory/stress responses in benign contexts *versus* hyperactivated immune networks melanoma—mirrors clinical-pathological transitions, suggesting these modules may encode critical switches governing the melanocytic nevi-to-melanoma transformation.

### 3.4 Homology analysis reveals complexity and instability in melanoma immune networks

Homology, a tool from algebraic topology, associates algebraic structures like groups, rings, or modules with topological spaces. It provides a framework for capturing features like “holes” in geometric shapes (such as connected components, loops, and voids). The GLMY homology theory, proposed by Shing-Tung Yau and colleagues, holds promise for evaluating melanoma immune-directed topological networks (Wu et al., 2023). Building on prior findings, we extended our analysis to examine the homology of both coarse-grained and fine-grained networks.

Under Betti-0 conditions, we found that benign samples contained more connected components (15 in total), with only one Betti-1 loop and no Betti-2 voids. In contrast, malignant samples exhibited the highest number of Betti-2 voids (112 in total), but far fewer lower-dimensional features (Supplementary Data S1). This pattern suggests that, in benign samples, gene networks tend to form multiple submodules that function in a relatively independent and stable manner, whereas in malignant samples, the immune module network appears more fragmented, with some modules potentially involved in unrelated biological functions. Additionally, the features in benign samples persisted for a longer duration during the filtration process, while those in malignant samples vanished more quickly, reflecting the instability of tumor immune networks. We also observed that the M29-associated filtration interval had the broadest coverage in the benign network, further underscoring its dominant role.

### 3.5 DNA repair and cell migration genes as hub module in melanoma immune network

We also conducted a homology analysis of the fine-grained networks within the modules and observed phenomena similar to those seen in the coarse-grained analysis. The internal gene networks of module 17 and module 29 exhibited significantly higher complexity in malignant groups, though with shorter filtration intervals (Supplementary Data S1). Furthermore, Betti-0 features (connected components) were relatively scarce in both benign and malignant groups, likely due to stronger interactions among genes within individual modules.

Based on enrichment analysis and a review of previous studies, we examined the main functions of genes within the M29 and M17 modules. M29 is a hub module in nevus group, with genes predominantly involved in cell cycle-related functions. These genes are crucial for regulating the cell cycle, ensuring proper cell division and proliferation, and affecting immune responses and cellular functions. AURKA ensures proper mitosis, CCNE1 drives the transition from G1 to S phase, and FOXM1 promotes G2/M progression. E2F1 controls DNA synthesis and S phase entry, while MAD2L1 and TTK (Mps1) ensure accurate chromosome segregation through the spindle checkpoint. UBE2S and UBE2T regulate cell cycle progression *via* the ubiquitin-proteasome system, ensuring genomic stability and effective immune function (Supplementary Figure S1, Supplementary Data S2).

In contrast, M17 is an active module in melanoma, with genes primarily associated with DNA damage and repair mechanisms, the Rho GTPase pathway, and immune cell migration. Genes like APEX2, RNF168, and RPA2 are involved in DNA repair, crucial for maintaining genomic stability in immune cells. ARHGAP24 and ARHGEF3 are linked to the Rho GTPase signaling pathway, which is vital for the activation, morphological changes, and migration of immune and non-immune cells. Additionally, genes such as DOCK2, ARHGAP24, and KCTD18 regulate cytoskeletal reorganization and cell migration, ensuring that immune cells effectively reach infection or inflammation sites (Supplementary Figure S1, Supplementary Data S2). Overall, these genes play significant roles in enhancing immune response and maintaining immune cell function.

### 3.6 The immune shift from nevi to melanoma is regulated by gene clusters involving AURKA and APEX2

**FIGURE 5:**
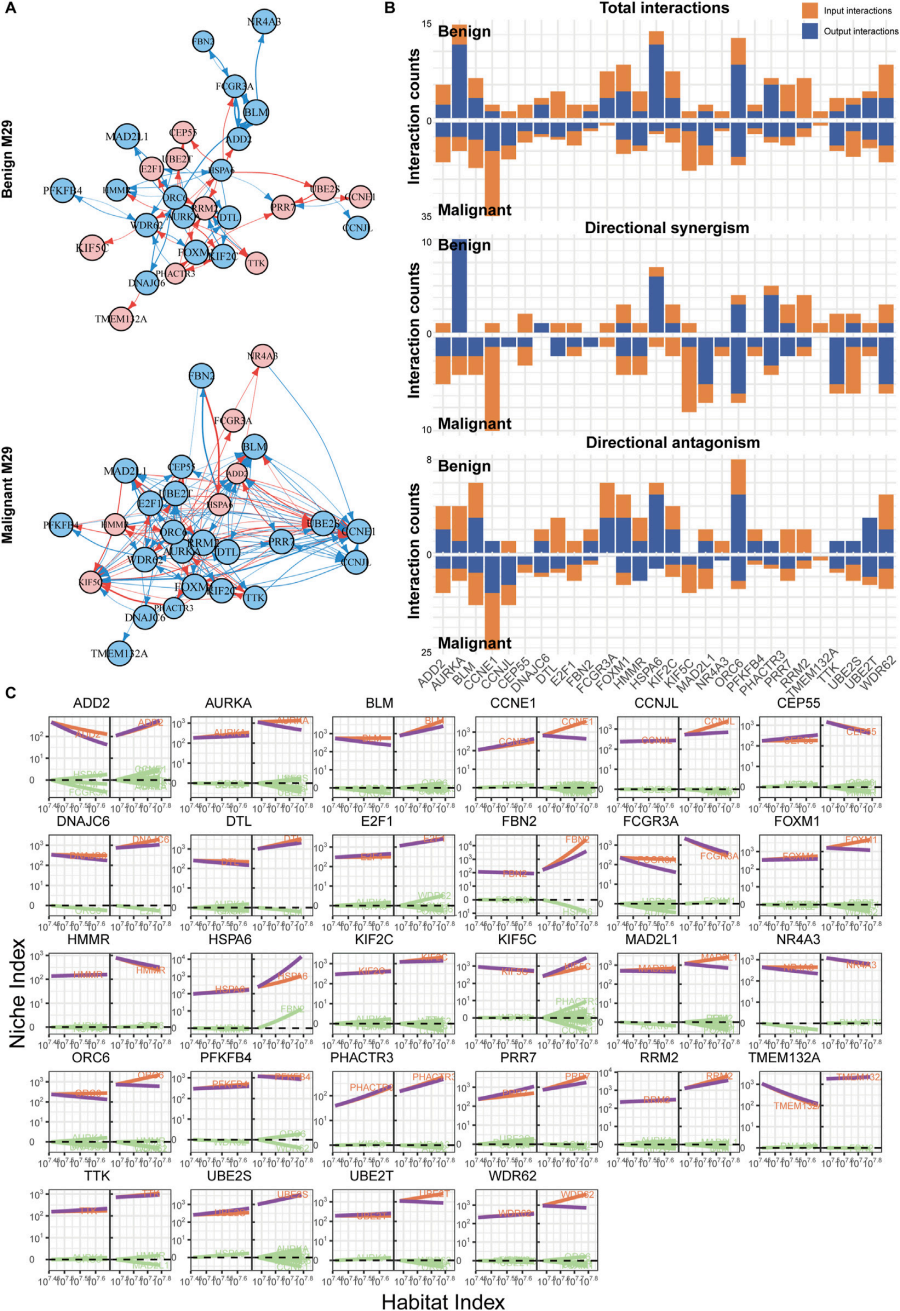
Immune network reconstruction of the hub module (M29) in nevi. **(A)** Fine-grained directed topological network within the M29 module. **(B)** Bar chart of the interaction relationships within the two groups’ networks. **(C)** Decomposition curve of each module’s NI based on the trend of HI changes.

Using a similar approach, we also reconstructed the networks for modules M17 and M29. M29, the hub module in the nevus network, exhibited a significant increase in the number of edges and regulatory intensity after malignancy, similar to the changes observed between modules (Figures 5A, B). In the benign group, the network was primarily controlled by AURKA, HSPA6, and ORC6, but the regulatory intensity was not strong (Figure 5C). In contrast, the malignant group’s network structure became more complex, with multiple genes, led by CCNE1, jointly regulating and displaying more antagonistic effects. Furthermore, in the malignant group, many genes showed steeper curves, indicating stronger allometric scaling and mutual regulation (Figure 5C).

**FIGURE 6:**
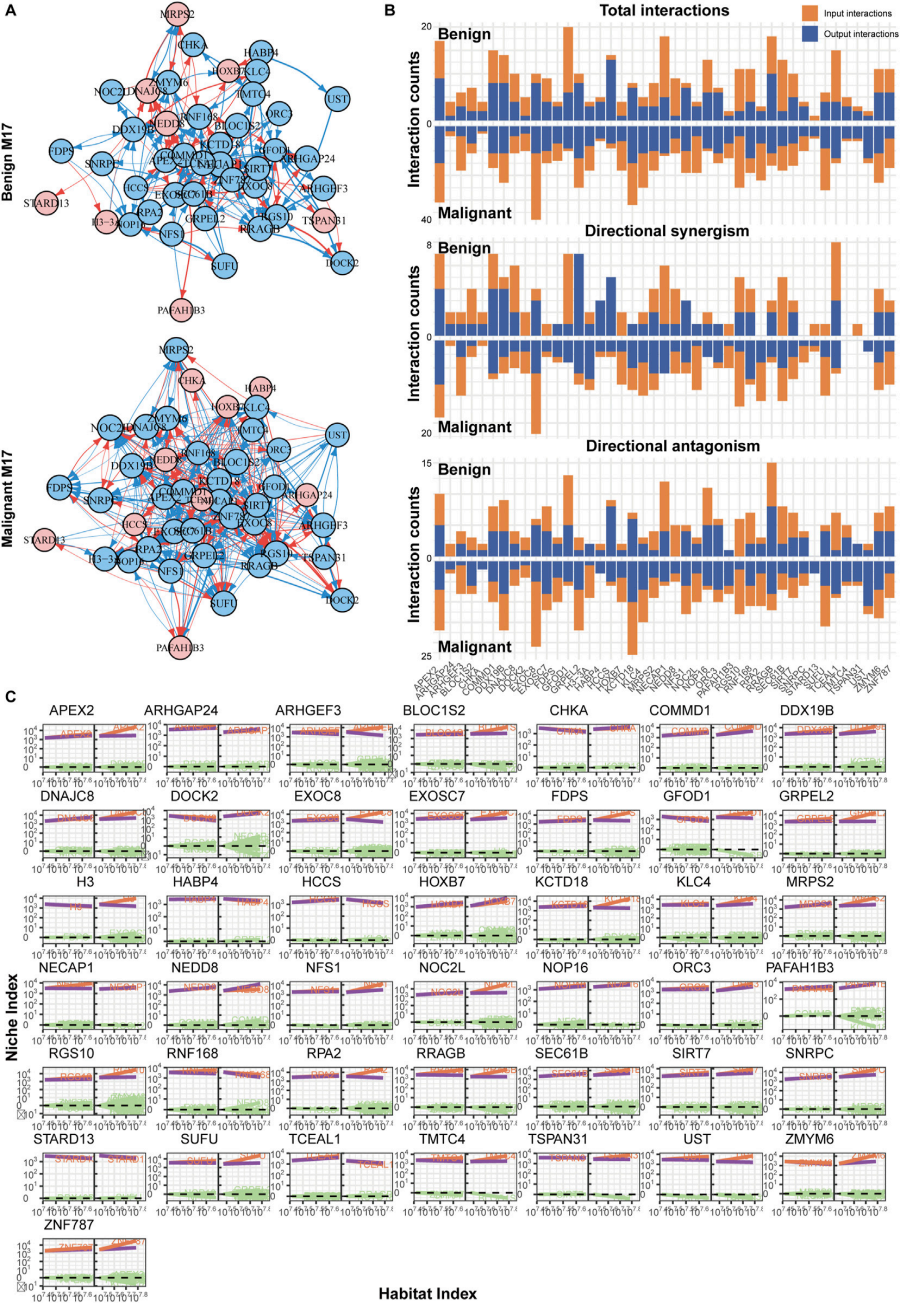
Immune network reconstruction of the hub module (M17) in melanoma. **(A)** Fine-grained directed topological network within the M17 module. **(B)** Bar chart of the interaction relationships within the two groups’ networks. **(C)** Decomposition curve of each module’s NI based on the trend of HI changes.

In comparison, the situation with M17 might be even more complex (Figure 6A). On one hand, M17 had a larger number of nodes; on the other hand, there were more active genes within M17. Their interactions were predominantly inhibitory, with FGOD1 being the most regulated, while HCCS and APEX2 exerted more regulation (Figure 6B). However, similar to what was previously described, in the benign group, although there were numerous interactions between nodes, the weights were relatively weak, and the impact on target genes might be limited (Figure 6C). This was also observed in modules like M1, M11, and M21 (Supplementary Figure S2–S4). In the malignant group, gene interactions became more frequent, and there was a lack of a significant hub gene, indicating a network determined by multiple genes. Most genes exhibited mutual antagonistic trends, with stronger regulatory intensities compared to the benign group (Figure 6C).

### 3.7 Drug screening using deep learning-based molecular docking models

To further explore the clinical value of potential targets identified through the topological network, we conducted drug-target interaction predictions using a deep learning model. A total of 15 structural prediction models were utilized, each incorporating 25,112 drug-target pairs to calculate the binding affinity of drug-target proteins. By selecting the top five scoring drug-target pairs in any model, 22drugs were identified as having potential therapeutic capabilities for melanoma (Table 1, Supplementary Data S3). Some of these drugs are already in phase III clinical trials or are currently in clinical use (e.g., imatinib, which is undergoing continuous clinical trials for repurposing) (Table 2). Almost all drugs have indications for malignant neoplastic diseases. Notably, some drugs exhibited binding affinity to multiple proteins, which might be attributed to the similar substructures of these proteins.

**TABLE 1:** Predicted drug-target binding score of top 22 drugs in 15 different models.

Drug name	Target	CNN_CNN_DAVIS	Morgan_CNN_DAVIS	MPNN_CNN_DAVIS	Daylight_AAC_DAVIS	Morgan_AAC_DAVIS	CNN_CNN_BindingDB	Morgan_CNN_BindingDB
AEG-41174	KLC4	4.97	5.08	10.47	5.06	5.24	6.65	6.36
ENMD-2076	ADD2	5.15	5.34	4.79	5.18	5.12	5.66	5.11
MLN-8054	AURKA	6.01	7.35	7.20	7.66	7.49	6.83	8.44
tozasertib lactate	BLOC1S2	5.04	5.08	5.09	5.42	5.02	5.86	5.21
IPH-1101	CHKA	4.91	5.07	4.47	5.06	5.16	6.97	4.28
imatinib	AURKA	6.59	5.06	4.11	5.06	4.95	4.92	4.98
tezacitabine	APEX2	5.02	5.10	4.45	5.07	5.93	5.11	4.31
fludarabine phosphate	DDX19B	4.89	5.06	36.03	5.07	5.68	5.09	3.40
nilotinib	DDX19B	5.47	5.06	40.75	5.09	4.97	4.95	5.12
imidazo[1,2-a] pyrazines	DDX19B	4.93	5.05	4.91	5.02	5.10	7.12	5.87
AG-957	AURKA	6.59	5.65	4.93	5.08	5.12	4.19	5.42
R-763	DOCK2	4.96	5.07	4.75	6.86	5.73	7.34	7.44
ABT-510	ARHGEF3	5.01	5.07	20.87	5.05	5.21	5.68	4.68
SUN-K954	AURKA	6.75	5.80	8.63	5.04	4.98	7.49	6.15
aurora A kinase inhibitors	AURKA	6.40	7.21	5.92	6.97	7.40	6.85	7.02
BAY-1217389	CHKA	4.98	5.13	7.60	5.39	5.11	7.92	6.25
alisertib	AURKA	5.48	7.08	7.08	6.78	7.32	5.91	7.12
luvixasertib	APEX2	4.88	5.07	8.26	5.08	5.18	8.39	7.09
tinengotinib	APEX2	4.89	5.12	3.52	5.08	5.21	7.14	4.31
risedronic acid sodium	DDX19B	5.09	5.03	43.72	5.45	5.17	5.61	5.15
plitidepsin	PAFAH1B3	4.95	5.06	5.10	5.03	5.03	7.28	6.00
olverembatinib	ARHGAP24	4.77	5.09	3.74	5.08	5.06	6.84	8.94

MPNN_CNN_BindingDB	Transformer_CNN_BindingDB	Daylight_AAC_BindingDB	Morgan_AAC_BindingDB	Morgan_CNN_KIBA	MPNN_CNN_KIBA	Daylight_AAC_KIBA	Morgan_AAC_KIBA
28.18	5.18	6.52	6.20	11.73	12.15	11.63	11.73
6.54	5.74	5.00	3.99	11.56	38.61	12.24	11.56
4.23	3.30	7.67	7.15	11.99	12.35	12.65	11.99
6.72	3.85	5.85	6.07	11.20	39.00	11.52	11.20
6.77	5.83	7.01	6.04	11.60	39.77	11.27	11.60
2.45	5.65	5.01	5.09	12.01	20.47	12.26	12.01
4.67	8.89	6.55	4.42	11.39	11.50	11.09	11.39
17.08	3.93	6.76	3.86	11.82	6.78	10.76	11.82
15.55	5.01	5.00	5.06	11.17	6.47	11.57	11.17
3.21	3.93	7.42	6.66	14.50	11.02	13.47	14.50
5.91	4.43	4.97	5.97	10.79	12.82	11.68	10.79
3.94	4.06	7.60	7.05	13.13	10.00	14.21	13.13
5.59	3.95	4.05	3.75	11.48	47.77	11.44	11.48
5.43	5.30	6.91	7.04	12.00	12.21	10.63	12.00
10.96	3.30	7.48	6.30	11.91	10.67	12.11	11.91
6.96	5.90	9.62	6.99	11.01	11.59	11.56	11.01
4.68	3.30	7.54	7.08	12.33	22.36	12.82	12.33
5.06	8.78	8.45	6.45	11.34	17.18	11.82	11.34
4.29	8.80	7.29	4.07	11.58	10.82	11.96	11.58
18.34	4.11	4.99	3.76	11.02	6.05	11.53	11.02
32.68	4.06	8.00	7.16	10.82	11.86	11.48	10.82
6.23	6.46	8.22	7.80	11.33	11.09	12.04	11.33

**TABLE 2:** Clinical data of interested drugs.

Drug Name	Mechanism of action	Drug disease	Highest status reached
ABT-510	Angiogenesis inhibitor; Thrombospondin 1 agonist, etc	Cancer	Phase II Clinical Trial
AEG-41174	Apoptosis stimulant; Bcr-Abl inhibitor; etc	Cancer, leukaemia	Phase I Clinical Trial
AG-957	Apoptosis stimulant; Bcr-Abl inhibitor	Cancer, unspecified	Preclinical
alisertib	Aurora kinase inhibitor; Mitotic inhibitor; etc	Cancer, leukaemia, lymphoma, myeloma, etc	Phase II Clinical Trial
aurora A kinase inhibitors	Aurora kinase inhibitor	Cancer, unspecified	Preclinical
BAY-1217389	TTK kinase inhibitor	Cancer, breast	Phase I Clinical Trial
ENMD-2076	Angiogenesis inhibitor; Aurora kinase inhibitor; etc	Cancer, leukaemia, lymphoma, myeloma, etc	Phase II Clinical Trial
fludarabine	DNA repair enzyme inhibitor; etc	Cancer, leukaemia, lymphoma, melanoma, etc	Phase III Clinical Trial
fludarabine phosphate	DNA repair enzyme inhibitor; DNA synthesis inhibitor; etc	Infection, HIV/AIDS	Preclinical
imidazo[1,2-a] pyrazines	Aurora kinase inhibitor	Cancer, unspecified	Preclinical
IPH-1101	Immunostimulant; T cell stimulant	Cancer, leukaemia, melanoma, Infection, etc	Phase II Clinical Trial
luvixasertib	TTK kinase inhibitor	Cancer, breast Cancer, solid, unspecified	Phase II Clinical Trial
MLN-8054	Aurora kinase inhibitor; Mitotic inhibitor; Protein kinase inhibitor	Cancer, colorectal, lung, sarcoma, unspecified, etc	Phase I Clinical Trial
nilotinib	Abl receptor tyrosine kinase inhibitor; Bcr-Abl inhibitor; C-kit inhibitor; etc	Cancer, unspecified	Preclinical
olverembatinib	Bcr-Abl inhibitor; C-kit inhibitor	Cancer, leukaemia, gastrointestinal, etc	Phase II Clinical Trial
plitidepsin	Apoptosis stimulant; Cell cycle inhibitor; CLN1 inhibitor; etc	Cancer, myeloma, lymphoma, Hodgkin’s, etc	Phase III Clinical Trial
R-763	Aurora kinase inhibitor; Mitotic inhibitor; Protein kinase inhibitor	Cancer, breast, colorectal, leukaemia, lymphoma, etc	Phase III Clinical Trial
risedronic acid sodium, delayed-release	Bisphosphonate; Bone resorption inhibitor; etc	Osteoporosis	Launched
SUN-K954	Bcr-Abl inhibitor	Cancer, leukaemia, chronic myelogenous, etc	Preclinical
tezacitabine	DNA inhibitor; DNA synthesis inhibitor; etc	Cancer, biliary, leukaemia, lung, oesophageal, etc	Phase II Clinical Trial
tinengotinib	Aurora kinase inhibitor; FGF receptor 1 tyrosine kinase inhibitor; etc	Cancer, biliary, breast, gastrointestinal, prostate, etc	Phase III Clinical Trial
tozasertib lactate	Aurora kinase inhibitor; Bcr-Abl inhibitor; Mitotic inhibitor; etc	Cancer, colorectal, leukaemia, lung, Myelodysplastic syndrome, etc	Phase II Clinical Trial

## 4 Discussion

Melanoma is a highly aggressive and potentially lethal type of skin cancer. Its danger stems from its rapid metastasis to other body parts, resulting in considerable morbidity and mortality. The molecular mechanisms underlying melanoma are complex, involving various genetic mutations, signaling pathways, and interactions with the tumor microenvironment. These intricate molecular processes contribute to the cancer’s resilience and ability to evade the immune system. As a result, treating melanoma is exceptionally challenging. Traditional therapies often fall short, and while new targeted therapies and immunotherapies offer hope, their effectiveness can be limited by the cancer’s ability to adapt and develop resistance. This complexity underscores the critical need for ongoing research to better understand melanoma’s biology and to develop more effective treatments.

Firstly, through directed topological network reconstruction, we observed significant changes in the immune gene regulatory network during the progression from benign melanocytic nevi to malignant melanoma. The transition from module M29 in benign nevi to module M17 in malignant melanoma highlights substantial shifts in tumor biology. M29, crucial in nevi, is primarily involved in cell cycle regulation. Upon malignant transformation, the key module changes to M17, indicating a shift in cell cycle regulation focus during tumor progression. This transition suggests major adjustments in cellular functions during malignancy. In melanoma, the M17 module is associated with DNA damage repair, the Rho GTPase pathway, and immune cell migration. This change may reflect the adaptation of malignant melanoma cells through enhanced DNA repair capabilities, altered cell migration patterns, and immune microenvironment modulation. These adaptations enable tumor cells to thrive and spread within the microenvironment. Furthermore, the functions of M17, related to immune cell migration and the tumor’s immune microenvironment, suggest that melanoma cells can evade host immune surveillance by modifying immune-related mechanisms. This enables better escape and dissemination of the tumor cells, illustrating the evolving strategies of malignancy in response to the immune system.

Our findings may also offer valuable guidance for clinical drug treatments of melanoma and could help elucidate some reasons behind the poor drug responses observed in certain cases. For instance, genes such as AURKA, APEX2, CCNE1, and FOXM1 have been reported to be associated with tumor resistance (Grunda et al., 2010; Quan et al., 2013; Zheng et al., 2023; Turner et al., 2019). Notably, their regulatory relationships within the melanoma immune network have undergone significant alterations (Figures 5, 6), indicating their potential role in melanoma drug resistance. However, further understanding of tumor resistance relies on specifically designed clinical data. For example, Mallardo et al. conducted a series of studies to explore factors influencing the efficacy of anti-PD1 therapy in melanoma across different grades (Mallardo et al., 2022a, 2022b; Mallardo et al., 2023; Mallardo et al., 2024). One of their studies collected clinical follow-up data, RNA sequencing, and proteomic data from melanoma patients treated with anti-PD1 drugs (Mallardo et al., 2024). The researchers employed LASSO regression to identify potential genes associated with responsiveness to anti-PD1 therapy, ultimately confirming five genes that were closely related to treatment sensitivity and patient progression-free survival (PFS). Topological analyses of sequencing data from resistant *versus* drug-sensitive samples will further aid in unraveling the mechanisms underlying drug resistance.

Secondly, using deep learning models, we have identified potential targeted drugs that could hold significant promise in the treatment of melanoma. These include various kinase inhibitors, DNA repair modulators, and immune modulators, targeting crucial molecular pathways involved in melanoma progression. For instance, drugs like imatinib and nilotinib target specific tyrosine kinases, which play a critical role in cell proliferation and survival. Aurora kinase inhibitors, such as MLN-8054 and alisertib, disrupt mitosis, leading to cell cycle arrest and apoptosis in melanoma cells. Additionally, drugs like fludarabine and plitidepsin modulate DNA repair mechanisms and enhance immune responses, respectively. Some of these drugs have shown potential efficacy against melanoma in experimental or early clinical trials, such as fludarabine, IPH-1101 and imatinib (Jiang et al., 2008; Vilgelm et al., 2015; Plummer et al., 2013). These compounds offer promising avenues for enhancing therapeutic efficacy, overcoming resistance, and potentially improving patient outcomes in melanoma treatment. By targeting multiple pathways, these drugs can work synergistically to inhibit tumor growth and metastasis, highlighting their potential as part of comprehensive melanoma treatment strategies. Further, as previously mentioned, several drugs exhibited binding affinity to multiple proteins. Some of these interactions had not been identified before, potentially addressing gaps in our understanding of the mechanisms of action of these drugs.

Thirdly, and most importantly, we introduce a new topology and deep learning-based paradigm for NGS data research. In the past decade, the cost of transcriptome sequencing has decreased by over 100-fold, making it comparable to or even less expensive than some common clinical tests such as blood biochemistry, MRI, and CT. Simultaneously, the precision and depth of transcriptome sequencing have significantly improved—under similar conditions, RNAseq demonstrates much higher performance than metagenomics (Hempel et al., 2022). Additionally, the sample size required for RNAseq has been continuously decreasing. A decade ago, RNAseq in laboratories often required a sample volume of 5 × 5 × 5 mm to ensure sufficient RNA extraction and identification. Currently, some commercial RNAseq services require only 1,000 cells for sequencing. Gene testing is now widely applied in clinical diagnosis, and RNAseq, with its higher potential for application, provides a novel non-invasive or minimally invasive approach for detecting lesion status or conducting microbial screening at the gene expression level. However, relying solely on a single analytical method may not adequately meet researchers’ needs. Traditional approaches, which calculate average expression levels or log fold changes of genes based on their FPKM values in samples, allow us to highlight genes that exhibit significant changes during pathological processes and identify potential biomarkers or targets. In contrast, topological methods shift the focus from revealing expression differences of individual genes or modules to emphasizing the regulatory relationships among multiple genes and modules. Our topological analysis uses NI and HI functional curves to describe the dynamic expression patterns of each gene across different conditions (benign or malignant). This approach highlights how gene or module expression fluctuates with habitat changes and enables us to calculate interactions between genes or modules. Consequently, some genes may not demonstrate the most pronounced changes in expression levels—such as those typically ranked in the top 10 or top 20—yet from a topological perspective, they can exert strong regulatory effects on the entire functional network. This broader approach has the potential to address gaps in traditional methods and enhance our understanding of gene interactions.

Simultaneously, numerous studies have advanced sequencing algorithm improvements through various technological approaches (Datlinger et al., 2021; Han et al., 2021). Many researchers have trained diagnostic models based on sequencing data using machine learning and large language model (LLM) methods (Shokhirev and Johnson, 2022; Elsborg and Salvatore, 2023). However, these models, based on “weak AI” (as nearly all current AI models are), often suffer from the “black box effect.” While they can accurately identify and classify data, their internal decision-making processes remain opaque, limiting our ability to understand specific pathological processes and discover therapeutic solutions. In contrast, reconstructing topological networks allows for a clearer observation of the logic behind gene network fluctuations. Topology offers a promising solution, with numerous excellent studies already leveraging topological methods to address research questions (Failmezger et al., 2020; Benjamin et al., 2024). However, most current topological models in use are undirected, making it difficult to observe regulatory relationships between different nodes. Directed topological models, on the other hand, enable us to understand these relationships, thereby enhancing our understanding of disease pathology and aiding in the interpretation of sequencing data for future clinical diagnostic applications.

We also utilized deep learning models for DTI (Drug-Target Interaction) prediction. Unlike clinical data analysis, AI-based deep learning is inherently suitable for *in silico* drug screening. As clinicians, we are often more interested in knowing which drugs can be used for targeted therapy, even if we do not yet understand the molecular binding mechanisms due to the “black box effect.” Clinical and preclinical drug screening is typically extremely expensive and time-consuming (costing billions of dollars and taking 5–10 years), and can occasionally encounter ethical issues (Jentzsch et al., 2023). *In silico* screening effectively addresses these problems and has already been widely applied in fundamental research in physics, chemistry, and biology (Wang et al., 2023). Meanwhile, previous drug development efforts heavily relied on human understanding and knowledge of the underlying mechanisms of the system being studied, which can be suboptimal and inefficient. AI based on deep learning can better fit the key parameters of complex systems and identify new breakthroughs.

Despite these insights, there are still areas where the level of topological analysis could be further enhanced. Firstly, although our dataset of 80 RNA-seq samples is relatively large, it remains limited, and expanding it could improve model accuracy. At the same time, our study focused exclusively on immune-related genes, but it would be intriguing to extend topological analyses to whole sequencing data or explore different formats, such as large-scale single-cell sequencing. Integrating multi-omics data, including genomics, transcriptomics, and proteomics, through layered analyses would also be a valuable approach. Moreover, incorporating time-series data, particularly large datasets that capture distinct stages of melanoma progression, could offer deeper insights into the development of the disease. However, these efforts would demand more powerful computational resources and more advanced model designs. Secondly, there remains room for further mathematical optimization in topological gene network analysis. For instance, the application of new algorithms for allometric scaling laws or other linear and nonlinear fitting methods could enhance our findings, as well as improve the interpretation of topological data. While our results are interpreted based on homology theory, numerous other algorithms exist for analyzing topological networks. For example, in social network analysis, metrics such as degree distribution and clustering coefficient can reveal social patterns among individuals and help understand how information spreads within groups. Community detection techniques are applied in fields like network security and sociology, while some protein interaction network models are also based on these theories. Random networks and small-world network models hold potential for interpreting neuronal activity. Incorporating diverse methods to parse sequencing data could lead to new discoveries, though additional work is necessary to connect this approach with clinical data. Lastly, while DTI predictions can evaluate the binding strength of known drugs, discovering more effective targeted treatments still depends on ongoing laboratory research and molecular structure predictions. We believe that further exploration in these areas will contribute significantly to our clinical and experimental research.

## Data Availability

This data can be found here: https://www.ncbi.nlm.nih.gov/geo/query/acc.cgi?acc=GSE112509.

## Abbreviations

AIC, Akaike Information Criterion; BIC, Bayesian Information Criterion; CNS, Central Nervous System; DTI, Drug–Target Interaction; FDA, Food and Drug Administration; HI, Habitat Index; ICI, Immune Checkpoint Inhibitors; NI, Niche Index; qdODE, Quasi-Dynamic system of Ordinary Differential Equations; SEER, Surveillance, Epidemiology, and End Results database; UV, Ultraviolet.

## References

[B1] Baller D. Thomas D. M. Cummiskey K. Bredlau C. Schwartz N. Orzechowski K. (2019). Gestational growth trajectories derived from a dynamic fetal-placental scaling law. J. R. Soc. Interface 16, 20190417. 10.1098/rsif.2019.0417 31662073 PMC6833314

[B2] Benjamin K. Bhandari A. Kepple J. D. Qi R. Shang Z. Xing Y. (2024). Multiscale topology classifies cells in subcellular spatial transcriptomics. Nature 630, 943–949. 10.1038/s41586-024-07563-1 38898271 PMC11208150

[B3] Bittencourt F. V. Marghoob A. A. Kopf A. W. Koenig K. L. Bart R. S. (2000). Large congenital melanocytic nevi and the risk for development of malignant melanoma and neurocutaneous melanocytosis. Pediatrics 106, 736–741. 10.1542/peds.106.4.736 11015516

[B4] Breuer K. Foroushani A. K. Laird M. R. Chen C. Sribnaia A. Lo R. (2013). InnateDB: systems biology of innate immunity and beyond–recent updates and continuing curation. Nucleic Acids Res. 41, D1228–D1233. 10.1093/nar/gks1147 23180781 PMC3531080

[B5] Brunsgaard E. K. Wu Y. P. Grossman D. (2023). Melanoma in skin of color: Part I. epidemiology and clinical presentation. J. Am. Acad. Dermatol 89, 445–456. 10.1016/j.jaad.2022.04.056 35533771

[B6] Chen C. Jiang L. Fu G. Wang M. Wang Y. Shen B. (2019). An omnidirectional visualization model of personalized gene regulatory networks. NPJ Syst. Biol. Appl. 5, 38. 10.1038/s41540-019-0116-1 31632690 PMC6789114

[B7] Cornish D. Holterhues C. Van De Poll-Franse L. V. Coebergh J. W. Nijsten T. (2009). A systematic review of health-related quality of life in cutaneous melanoma. Ann. Oncol. 20 (Suppl 6), vi51–vi58. 10.1093/annonc/mdp255 19617298 PMC2712593

[B8] Datlinger P. Rendeiro A. F. Boenke T. Senekowitsch M. Krausgruber T. Barreca D. (2021). Ultra-high-throughput single-cell RNA sequencing and perturbation screening with combinatorial fluidic indexing. Nat. Methods 18, 635–642. 10.1038/s41592-021-01153-z 34059827 PMC7612019

[B9] Dong A. Wu S. Che J. Wang Y. Wu R. (2023). idopNetwork: a network tool to dissect spatial community ecology. Methods Ecol. Evol. 14, 2272–2283. 10.1111/2041-210x.14172

[B10] Elder D. E. Bastian B. C. Cree I. A. Massi D. Scolyer R. A. (2020). The 2018 World Health Organization classification of cutaneous, mucosal, and uveal melanoma: detailed analysis of 9 distinct subtypes defined by their evolutionary pathway. Arch. Pathol. Lab. Med. 144, 500–522. 10.5858/arpa.2019-0561-RA 32057276

[B11] Elsborg J. Salvatore M. (2023). Using LLMs and explainable ML to analyze biomarkers at single-cell level for improved understanding of diseases. Biomolecules 13, 1516. 10.3390/biom13101516 37892198 PMC10605495

[B12] Failmezger H. Muralidhar S. Rullan A. De Andrea C. E. Sahai E. Yuan Y. (2020). Topological tumor graphs: a graph-based spatial model to infer stromal recruitment for immunosuppression in melanoma histology. Cancer Res. 80, 1199–1209. 10.1158/0008-5472.CAN-19-2268 31874858 PMC7985597

[B13] Grunda J. M. Fiveash J. Palmer C. A. Cantor A. Fathallah-Shaykh H. M. Nabors L. B. (2010). Rationally designed pharmacogenomic treatment using concurrent capecitabine and radiotherapy for glioblastoma; gene expression profiles associated with outcome. Clin. Cancer Res. 16, 2890–2898. 10.1158/1078-0432.CCR-09-3151 20460474 PMC2871063

[B14] Hamid O. Robert C. Daud A. Hodi F. S. Hwu W. J. Kefford R. (2019). Five-year survival outcomes for patients with advanced melanoma treated with pembrolizumab in KEYNOTE-001. Ann. Oncol. 30, 582–588. 10.1093/annonc/mdz011 30715153 PMC6503622

[B15] Han M. Liu X. Zhang W. Wang M. Bu W. Chang C. (2021). TSMiner: a novel framework for generating time-specific gene regulatory networks from time-series expression profiles. Nucleic Acids Res. 49, e108. 10.1093/nar/gkab629 34313778 PMC8502000

[B16] Hempel C. A. Wright N. Harvie J. Hleap J. S. Adamowicz S. J. Steinke D. (2022). Metagenomics versus total RNA sequencing: most accurate data-processing tools, microbial identification accuracy and perspectives for ecological assessments. Nucleic Acids Res. 50, 9279–9293. 10.1093/nar/gkac689 35979944 PMC9458450

[B17] Huang K. Fu T. Glass L. M. Zitnik M. Xiao C. Sun J. (2021). DeepPurpose: a deep learning library for drug-target interaction prediction. Bioinformatics 36, 5545–5547. 10.1093/bioinformatics/btaa1005 33275143 PMC8016467

[B18] Jentzsch V. Osipenko L. Scannell J. W. Hickman J. A. (2023). Costs and causes of oncology drug attrition with the example of insulin-like growth factor-1 receptor inhibitors. JAMA Netw. Open 6, e2324977. 10.1001/jamanetworkopen.2023.24977 37505498 PMC10383012

[B19] Jiang X. Zhou J. Yuen N. K. Corless C. L. Heinrich M. C. Fletcher J. A. (2008). Imatinib targeting of KIT-mutant oncoprotein in melanoma. Clin. Cancer Res. 14, 7726–7732. 10.1158/1078-0432.CCR-08-1144 19047099

[B20] Kalaora S. Nagler A. Wargo J. A. Samuels Y. (2022). Mechanisms of immune activation and regulation: lessons from melanoma. Nat. Rev. Cancer 22, 195–207. 10.1038/s41568-022-00442-9 35105962

[B21] Kim B. R. Zhang L. Berg A. Fan J. Wu R. (2008). A computational approach to the functional clustering of periodic gene-expression profiles. Genetics 180, 821–834. 10.1534/genetics.108.093690 18780724 PMC2567383

[B22] Kunz M. Löffler-Wirth H. Dannemann M. Willscher E. Doose G. Kelso J. (2018). RNA-seq analysis identifies different transcriptomic types and developmental trajectories of primary melanomas. Oncogene 37, 6136–6151. 10.1038/s41388-018-0385-y 29995873

[B23] Lehmann J. Dunbar R. I. (2009). Network cohesion, group size and neocortex size in female-bonded Old World primates. Proc. Biol. Sci. 276, 4417–4422. 10.1098/rspb.2009.1409 19793756 PMC2817113

[B24] Lo J. A. Fisher D. E. (2014). The melanoma revolution: from UV carcinogenesis to a new era in therapeutics. Science 346, 945–949. 10.1126/science.1253735 25414302 PMC4701046

[B25] Mallardo D. Fordellone M. White A. Vowinckel J. Bailey M. Sparano F. (2024). A combined proteomic and transcriptomic signature is predictive of response to anti-PD-1 treatment: a retrospective study in metastatic melanoma patients. Int. J. Mol. Sci. 25, 9345. 10.3390/ijms25179345 39273294 PMC11395026

[B26] Mallardo D. Simeone E. Vanella V. Vitale M. G. Palla M. Scarpato L. (2022b). Concomitant medication of cetirizine in advanced melanoma could enhance anti-PD-1 efficacy by promoting M1 macrophages polarization. J. Transl. Med. 20, 436. 10.1186/s12967-022-03643-w 36180872 PMC9523893

[B27] Mallardo D. Woodford R. Menzies A. M. Zimmer L. Williamson A. Ramelyte E. (2023). The role of diabetes in metastatic melanoma patients treated with nivolumab plus relatlimab. J. Transl. Med. 21, 753. 10.1186/s12967-023-04607-4 37880788 PMC10601323

[B28] Mallardo D. Giannarelli D. Vitale M. G. Galati D. Trillò G. Esposito A. (2022a). Nivolumab serum concentration in metastatic melanoma patients could be related to outcome and enhanced immune activity: a gene profiling retrospective analysis. J. Immunother. Cancer 10, e005132. 10.1136/jitc-2022-005132 36424033 PMC9693654

[B29] Neale H. Plumptre I. Belazarian L. Wiss K. Hawryluk E. B. (2022). Central nervous system magnetic resonance imaging abnormalities and neurologic outcomes in pediatric patients with congenital nevi: a 10-year multi-institutional retrospective study. J. Am. Acad. Dermatol 87, 1060–1068. 10.1016/j.jaad.2022.05.062 35716834

[B30] National Institutes of Health (2024). Cancer stat facts: melanoma of the skin. Available at: https://seer.cancer.gov/statfacts/html/melan.html (Accessed June 1, 2024).

[B31] Patel S. P. Othus M. Chen Y. Wright Jr G. P. Yost K. J. Hyngstrom J. R. (2023a). Neoadjuvant–adjuvant or adjuvant-only pembrolizumab in advanced melanoma. New England J. Med. 388, 813–823. 10.1056/NEJMoa2211437 36856617 PMC10410527

[B32] Patel V. R. Roberson M. L. Pignone M. P. Adamson A. S. (2023b). Risk of mortality after a diagnosis of melanoma *in situ* . JAMA Dermatol 159, 703–710. 10.1001/jamadermatol.2023.1494 37285145 PMC10248809

[B33] Plummer R. Lorigan P. Brown E. Zaucha R. Moiseyenko V. Demidov L. (2013). Phase I-II study of plitidepsin and dacarbazine as first-line therapy for advanced melanoma. Br. J. Cancer 109, 1451–1459. 10.1038/bjc.2013.477 23989947 PMC3776988

[B34] Quan M. Wang P. Cui J. Gao Y. Xie K. (2013). The roles of FOXM1 in pancreatic stem cells and carcinogenesis. Mol. Cancer 12, 159. 10.1186/1476-4598-12-159 24325450 PMC3924162

[B35] Rosenberg A. R. Weston S. J. Deshields T. Fields R. C. Linette G. P. Cornelius L. A. (2021). Health-related quality of life in patients with malignant melanoma by stage and treatment status. J. Am. Acad. Dermatol 85, 486–489. 10.1016/j.jaad.2018.06.007 29906543

[B36] Shokhirev M. N. Johnson A. A. (2022). An integrative machine-learning meta-analysis of high-throughput omics data identifies age-specific hallmarks of Alzheimer’s disease. Ageing Res. Rev. 81, 101721. 10.1016/j.arr.2022.101721 36029998

[B37] Siegel R. L. Miller K. D. Fuchs H. E. Jemal A. (2021). Cancer statistics, 2021. CA Cancer J. Clin. 71, 7–33. 10.3322/caac.21654 33433946

[B38] Spagnolo F. Boutros A. Tanda E. Queirolo P. (2019). The adjuvant treatment revolution for high-risk melanoma patients. Semin. Cancer Biol. 59, 283–289. 10.1016/j.semcancer.2019.08.024 31445219

[B39] Sung H. Ferlay J. Siegel R. L. Laversanne M. Soerjomataram I. Jemal A. (2021). Global cancer statistics 2020: GLOBOCAN estimates of incidence and mortality worldwide for 36 cancers in 185 countries. CA Cancer J. Clin. 71, 209–249. 10.3322/caac.21660 33538338

[B40] Turner N. C. Liu Y. Zhu Z. Loi S. Colleoni M. Loibl S. (2019). Cyclin E1 expression and palbociclib efficacy in previously treated hormone receptor-positive metastatic breast cancer. J. Clin. Oncol. 37, 1169–1178. 10.1200/JCO.18.00925 30807234 PMC6506420

[B41] Vilgelm A. E. Pawlikowski J. S. Liu Y. Hawkins O. E. Davis T. A. Smith J. (2015). Mdm2 and aurora kinase a inhibitors synergize to block melanoma growth by driving apoptosis and immune clearance of tumor cells. Cancer Res. 75, 181–193. 10.1158/0008-5472.CAN-14-2405 25398437 PMC4286469

[B42] Wang H. Fu T. Du Y. Gao W. Huang K. Liu Z. (2023). Scientific discovery in the age of artificial intelligence. Nature 620, 47–60. 10.1038/s41586-023-06221-2 37532811

[B43] Welch H. G. Mazer B. L. Adamson A. S. (2021). The rapid rise in cutaneous melanoma diagnoses. New England J. Med. 384, 72–79. 10.1056/NEJMsb2019760 33406334

[B44] Wu S. Liu X. Dong A. Gragnoli C. Griffin C. Wu J. (2023). The metabolomic physics of complex diseases. Proc. Natl. Acad. Sci. U. S. A. 120, e2308496120. 10.1073/pnas.2308496120 37812720 PMC10589719

[B45] Zheng D. Li J. Yan H. Zhang G. Li W. Chu E. (2023). Emerging roles of Aurora-A kinase in cancer therapy resistance. Acta Pharm. Sin. B 13, 2826–2843. 10.1016/j.apsb.2023.03.013 37521867 PMC10372834

[B46] Zhou X. Yang M. Liu Z. Li P. Xie B. Peng C. (2021). Dynamic allometric scaling of tree biomass and size. Nat. Plants 7, 42–49. 10.1038/s41477-020-00815-8 33398156

[B47] Zhou Y. Zhou B. Pache L. Chang M. Khodabakhshi A. H. Tanaseichuk O. (2019). Metascape provides a biologist-oriented resource for the analysis of systems-level datasets. Nat. Commun. 10, 1523. 10.1038/s41467-019-09234-6 30944313 PMC6447622

