# Supplementary material for: Identification of biomarkers and target drugs for melanoma: a topological and deep learning approach
Source: Front Genet. 2025 Mar 3;16:1471037. doi: 10.3389/fgene.2025.1471037 (PMC11911340; doi:10.3389/fgene.2025.1471037)
Supplement: Supplementary file 2 [file Table1.docx]

Supplementary Material

# Supplementary Data

Supplementary Data files have been uploaded separately.

Supplementary Data 1. Homology data of M17, M29 and whole network.

Supplementary Data 2. Metadata of modules.

Supplementary Data 3. DTI result of selected targets.

# Supplementary Figures


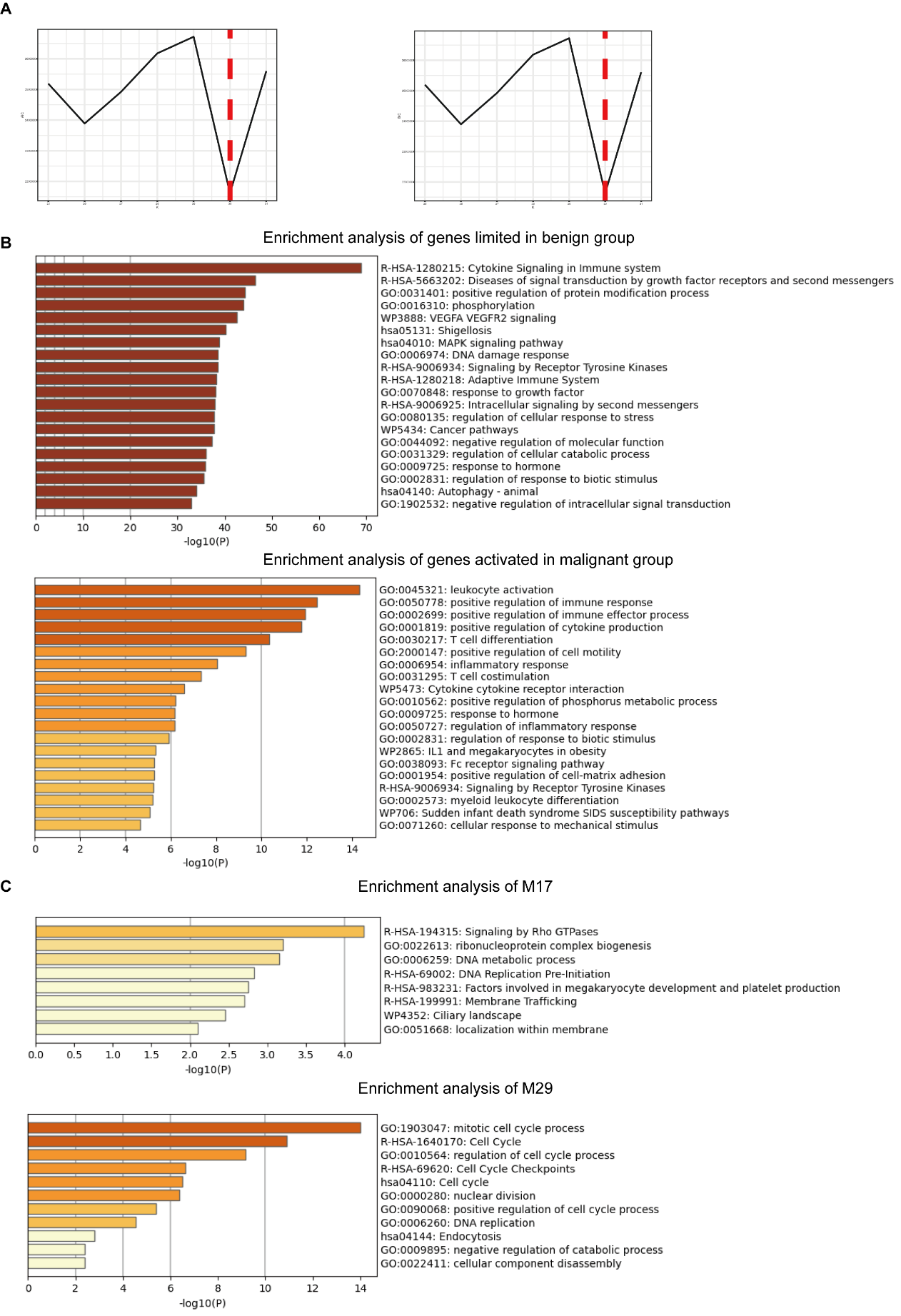


**Supplementary Figure 1.** Functional Clustering Analysis. A. Trends of AIC and BIC for functional clustering, with the lowest points indicated by red dashed lines. B. Functional enrichment results for modules with altered interaction patterns. C. Functional enrichment results for the two hub modules.
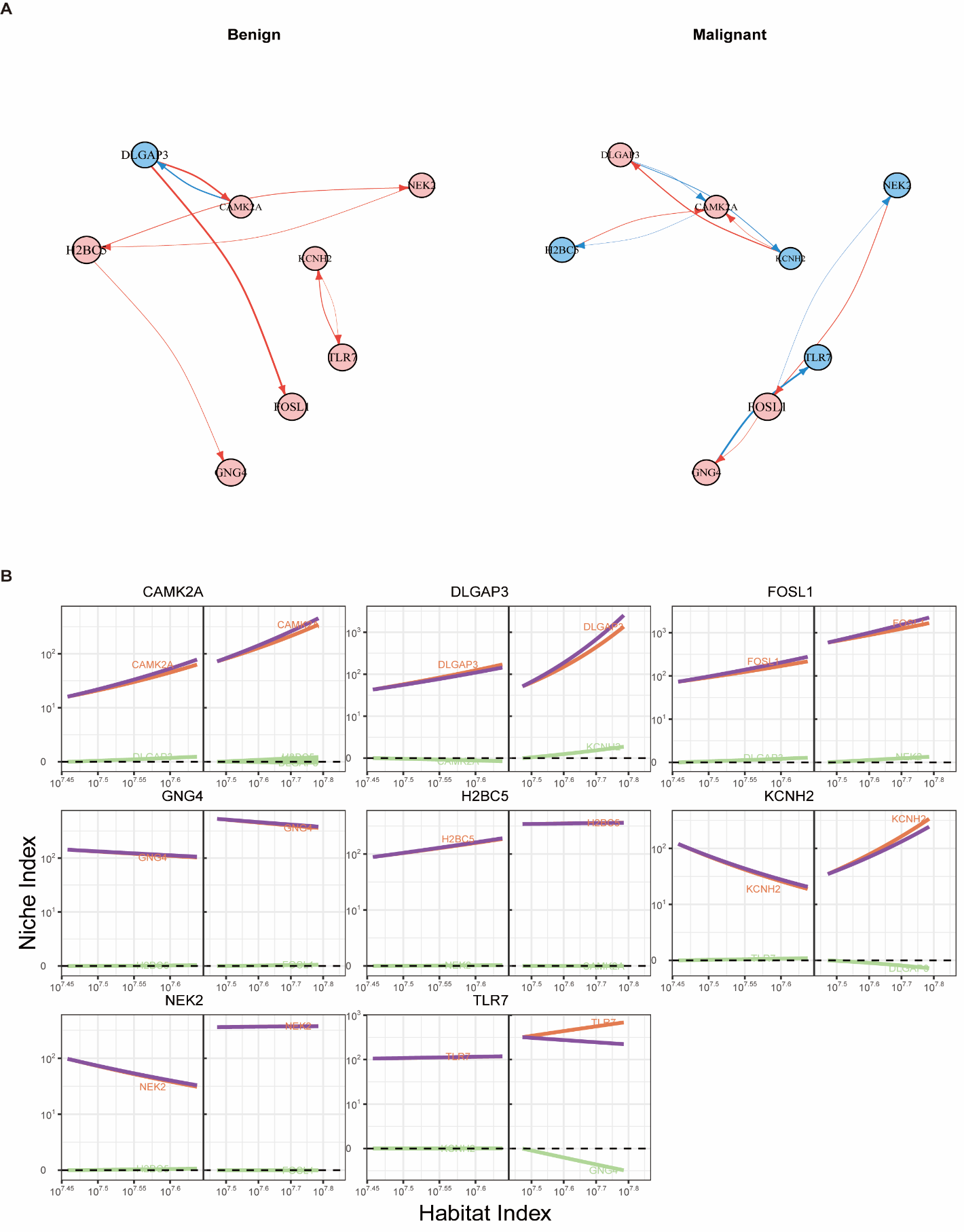
**Supplementary Figure 2.** Immune Network Reconstruction of Module M1 in Nevi and Melanoma. A. Regulatory network of Module M1. B. qdODE calculations for genes within Module M1.


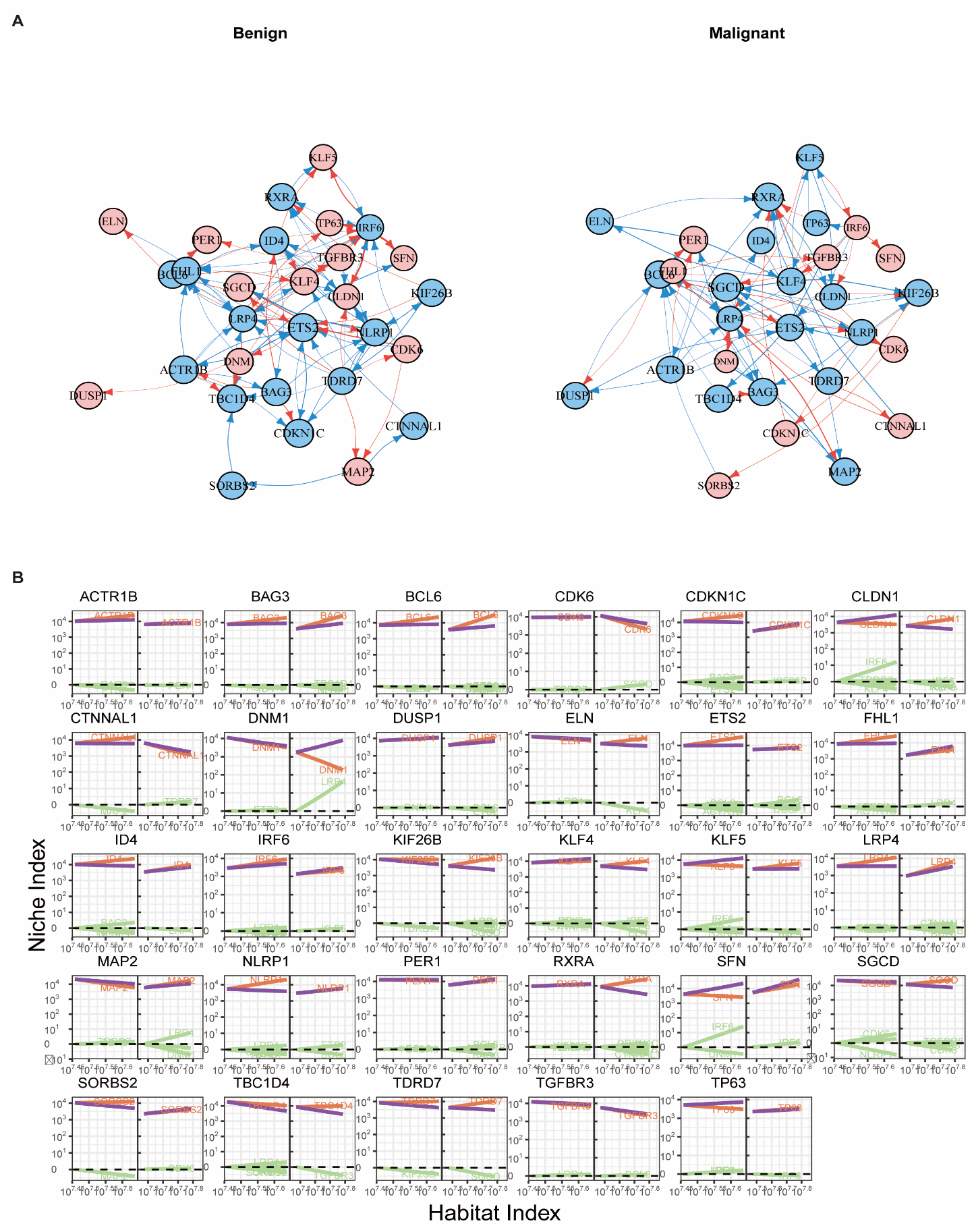


**Supplementary Figure 3.** Immune Network Reconstruction of Module M11 in Nevi and Melanoma. A. Regulatory network of Module M11. B. qdODE calculations for genes within Module M11.


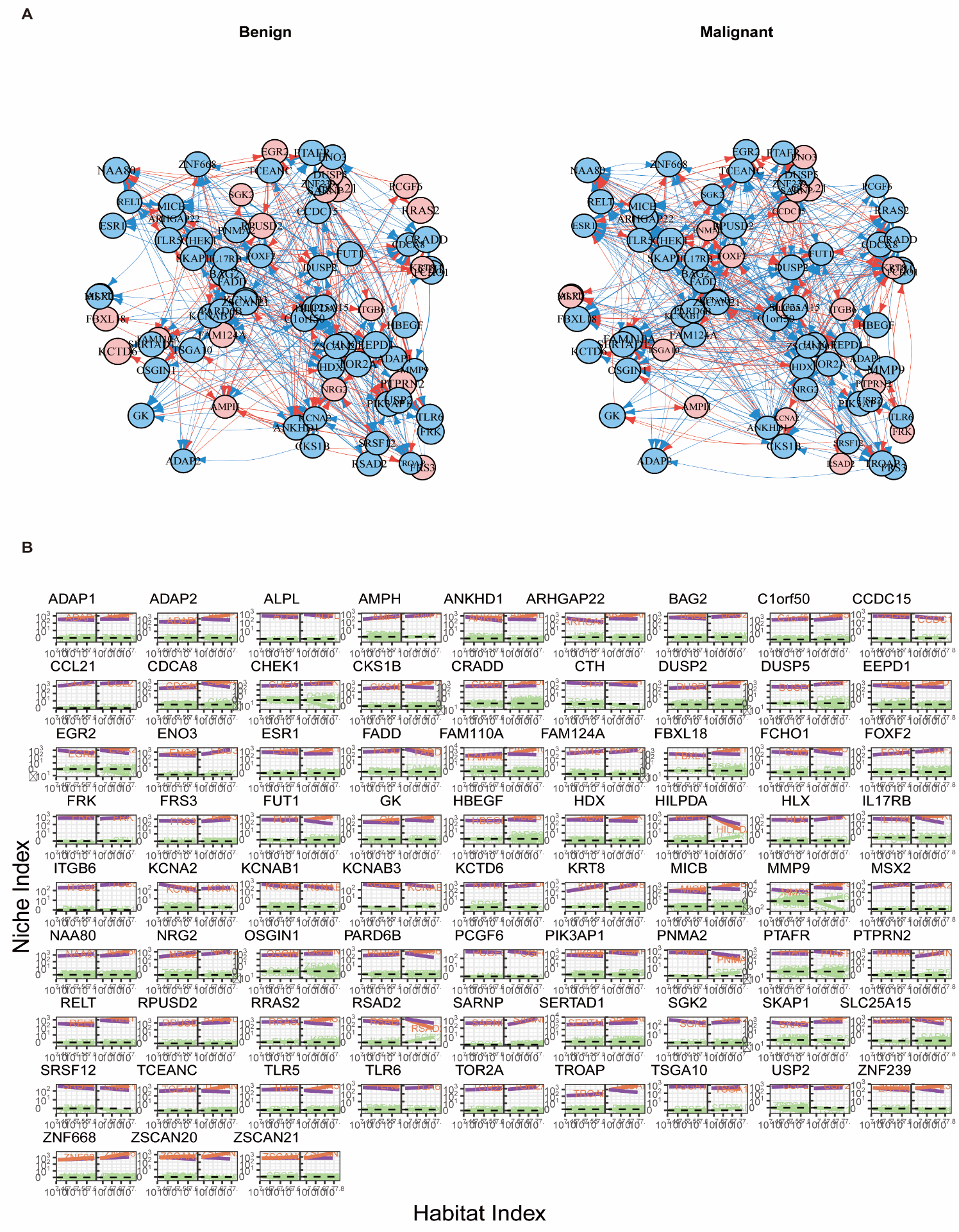


**Supplementary Figure 4.** Immune Network Reconstruction of Module M21 in Nevi and Melanoma. A. Regulatory network of Module M21. B. qdODE calculations for genes within Module M21.
